# Surgical trauma‐induced immunosuppression in cancer: Recent advances and the potential therapies

**DOI:** 10.1002/ctm2.24

**Published:** 2020-04-29

**Authors:** Fan Tang, Yan Tie, Chongqi Tu, Xiawei Wei

**Affiliations:** ^1^ State Key Laboratory of Biotherapy and Cancer Center West China Hospital Sichuan University Chengdu Sichuan People's Republic of China; ^2^ Department of Orthopeadics West China Hospital Sichuan University Chengdu Sichuan People's Republic of China; ^3^ Sichuan Cancer Hospital & Institute, Sichuan Cancer Center, School of Medicine University of Electronic Science and Technology of China Chengdu Sichuan People's Republic of China

**Keywords:** damage‐associated molecular pattern, postoperative immunosuppression, solid cancers, surgical trauma

## Abstract

Surgical resection remains the mainstay treatment for solid cancers, especially for localized disease. However, the postoperative immunosuppression provides a window for cancer cell proliferation and awakening dormant cancer cells, leading to rapid recurrences or metastases. This immunosuppressive status after surgery is associated with the severity of surgical trauma since immunosuppression induced by minimally invasive surgery is less than that of an extensive open surgery. The systemic response to tissue damages caused by surgical operations and the subsequent wound healing induced a cascade alteration in cellular immunity. After surgery, patients have a high level of circulating damage‐associated molecular patterns (DAMPs), triggering a local and systemic inflammation. The inflammatory metrics in the immediate postoperative period was associated with the prognosis of cancer patients. Neutrophils provide the first response to surgical trauma, and the production of neutrophil extracellular traps (NETs) promotes cancer progression. Activated macrophage during wound healing presents a tumor‐associated phenotype that cancers can exploit for their survival advantage. In addition, the amplification and activation of myeloid‐derived suppressor cells (MDSCs), regulatory T cells (Tregs) or the elevated programmed death ligand‐1 and vascular endothelial growth factor expression under surgical trauma, exacerbate the immunosuppression and favor of the formation of the premetastatic niche. Therapeutic strategies to reduce the cellular immunity impairment after surgery include anti‐DAMPs, anti‐postoperative inflammation or inflammatory/pyroptosis signal, combined immunotherapy with surgery, antiangiogenesis and targeted therapies for neutrophils, macrophages, MDSCs, and Tregs. Further, the application of enhanced recovery after surgery also has a feasible outcome for postoperative immunity restoration. Overall, current therapies to improve the cellular immunity under the special condition after surgery are relatively lacking. Further understanding the underlying mechanisms of surgical trauma‐related immunity dysfunction, phenotyping the immunosuppressive cells, and developing the related therapeutic intervention should be explored.

**Abbreviations**
ARG1: Arginase‐1
CCLs: C‐C motif chemokines
COX‐1 or COX‐2: cyclooxygenase 1 or 2
CRP: C‐reactive protein
CSF1: colony‐stimulating factor‐1
CSF‐1R: colony‐stimulating factor‐1 receptor
CXCLs: C‐X‐C motif chemokines
DAMPs,damage‐associated: damage‐associated molecular patterns
ERAS,enhanced recovery after: enhanced recovery after surgery
GITR: glucocorticoid‐induced TNFR‐related protein
G‐MDSC: granulocytic‐MDSC
HMGB1: high bobility group Box 1
HSP70: heat shock protein 70
HVEM: herpes virus entry mediator
IFN‐γ: interferon‐γ
IGF I and II: insulin‐like growth factor
IL‐6: interleukin‐6
IL‐8: interleukin‐8
MDSCs: myeloid‐derived suppressor cells
M‐MDSCs: monocytic‐MDSCs
mtDNA: mitochondrial DNA
NETs: neutrophil extracellular traps
NF‐κB: nuclear factor kappa‐B
NK cells: nature kill cells
NLRP3: NOD‐like receptor family, pyrin domain containing 3
NLRs: NOD‐like receptors
NSAIDs: nonsteroidal anti‐inflammatory drugs
PD‐1: programmed death‐1
PDGF: platelet derived growth factor
PD‐L1: programmed death ligand‐1
PGE‐2: prostaglandin E2
PI3Kδ: phosphatidylinositol 3‐kinase δ
PRR: pattern recognition receptors
TGF‐α: transforming growth factor
TGF‐β: transforming growth factor
TLRs: Toll‐like receptors
TNF‐α: tumor necrosis factor alpha
Tregs: regulatory T cells
VEGF: vascular endothelial growth factor

## 1 BACKGROUND

Surgical resection is currently the mainstay treatment for solid cancers. However, even received curative resection, postoperative recurrence remains high at 20–66%.^1^, ^2^ And metastases are the cause of most cancer‐related mortalities after resection.^3^ These clinical evidences indicated surgical resection tend to correlate with the metastatic seeding or proliferation of tumor cells. Surgical operations on solid tumors disseminate circulating tumor cells,^4^ which the sterile inflammation after surgery captures thereby supporting their survival and metastatic growth.^5^ Besides this, the cellular immunity is proved to be impaired under surgical stress that promotes the proliferation of tumor cells. The secretion of interferon‐γ (IFN‐γ), a cytokine that is integral in controlling metastases, is suppressed following colorectal cancer surgery.^6^ In addition, the total number of CD8^+^ T cells reduced after surgery, which finally promotes the outgrowth of implanted tumors.^7^ Up to now, the postoperative immunosuppression state has been clinically confirmed in surgeries involving thoracic cancers,^8^, ^9^ renal cell carcinoma,^10^ gastric cancer,^11^ colorectal cancer,^6^ pancreas adenocarcinoma,^12^ breast cancer,^13^ ovarian cancer,^14^ prostate cancer,^15^ and sarcoma^16^ (Table 1).

**TABLE 1:** Clinical studies about the immunity dysfunction after surgeries for solid cancers

Cancer types	Surgical strategies	The immunity dysfunction after surgery	The prognostic value	Reference
Lung cancer	Video‐assisted thoracoscopic surgery (VATS) versus open resection	VATS was associated with less effect on circulating CD4^+^ T cells at 2 days, on NK lymphocytes at 7 days postsurgery, lymphocyte oxidation suppression at 2 days.	‐	^8^
Colorectal cancer	Open and Laparoscopic	NK cell IFN‐γ secretion is significantly suppressed for up to 2 months following surgery.	‐	^6^
Prostate cancer	Radical prostatectomy	CD14^−^HLA‐DR^−^CD33^+^CD11b^+^ cells wereincreased.	‐	^15^
Breast cancer	Radical mastectomy	Peripheral FOXP3 mRNA level and Treg frequencies were elevated on postoperative day 7.	‐	^13^
Ovarian cancer	Debulking surgery	The levels of IL‐10 decreased after surgery.	Gal‐1 and CCL2 are independent prognostic factors for progression‐free survival and overall survival.	^14^
Gastric cancer	Minimally invasive surgery and Roux‐en‐Y gastric bypass	Differences within the open group were seen for T lymphocytes, NK cells, T‐helper lymphocytes, and CD4/CD8 subsets, significant decreases were found in cytotoxicity on day 1 and 2.	‐	^11^
Esophagus cancer	Esophagectomy	A thoracoscopic approach was a significant factor in attenuating IL‐6 and IL‐8 levels on postoperative day 1, and a longer operative time was a significant factor in increasing these levels.	‐	^9^
Pancreas adenocarcinoma	Curative pancreatectomy	The immunologic statusdeteriorated within 3 to 4 days after the operation and recovered after that.	Elevated neutrophil‐to‐lymphocyte ratio at postoperative 1 and 6 months and decreased total lymphocyte count at postoperative 1 month were significant prognosis predictors.	^12^
Osteosarcoma	Wide excision	The serum levels of VEGF and endostatin decreased after removal of the tumor.	The postoperative levels of VEGF, VEGF/platelets, and endostatin significantly higher in the recurrence group than the no‐recurrence group.	^16^
Renal cancer	Radical nephrectomy or nephron‐sparing surgery	Naïve T‐cells, memory T‐cells, CD16^+^ NK and total circulating dendritic cells worsened after 12 and 24 h from surgery.	‐	^10^

Surgery‐related factors such as sympathetic nervous system activation, blood transfusion, and anesthetic agents induce a postoperative immunosuppression. Clinical evidence indicated that the immunosuppressive effect after surgery seems to be associated with the invasiveness of surgery.^17^, ^18^, ^19^ Surgical intervention for solid cancers accompanied by unintended soft tissue damages have previously been reported.^20^ And open organ surgical operations accompanied by extensive tissue dissection produced a cellular immunity suppression, which was not observed in less invasive procedures.^21^ In colorectal cancer, open resection resulted in a greater immunity impairment that was associated with shorter disease‐free intervals and time‐to‐recurrence compared with laparoscopic resection.^22^ Reasons underling the relation of cellular immunity loss and the invasiveness of surgery are not clear. But these evidences put the surgical trauma on the central role of the possible reasons.

**FIGURE 1:**
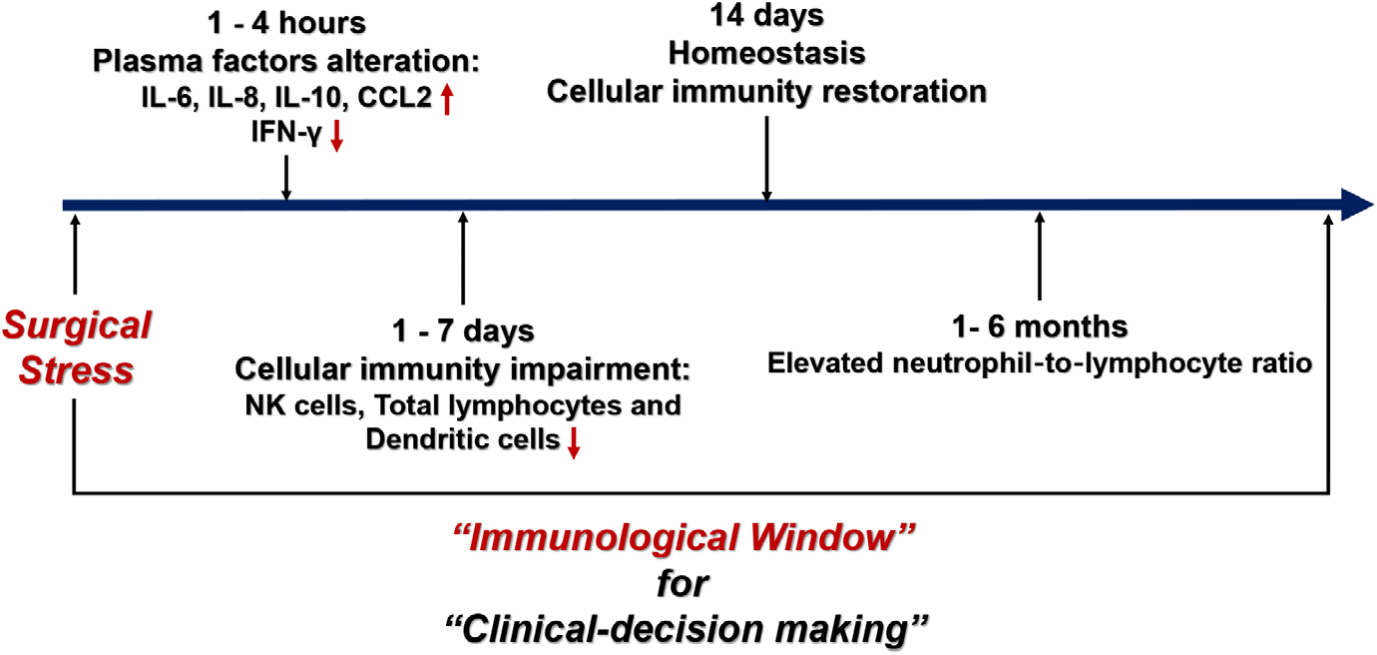
The timeline of the immunity dysfunction after surgical stress. Surgical stress disrupted the innate and adoptive immunity. The levels of proinflammation factors like IL‐6, IL‐8, IL‐10, CCL2, etc increased, and the level of cytokine IFN‐γ, which is secreted by NK cells and T lymphocytes, decreased in several hours after surgery. The activity of NK cells and the count of total lymphocytes and dendritic cells get impaired, and this immunosuppressive status commonly last 1 week after surgery. Due to the homeostasis of our body, the cellular immunity will get restored within 14 days after surgery. Recent clinical evidence revealed that the impairment of cellular immunity may last up to 6 months

The immunity suppressive state after surgery may last from several days to 6 months. The surgical intervention disrupts the homeostatic balance of the body, and the plasma levels of cytokine IFN‐γ begin to drop just 1 hour after surgery.^23^ In addition, the impairment of T cells proliferation and the activity of nature killer (NK) cells span for about 2 weeks, with each peaking on the fifth to– seventh day after surgery.^24^ The suppressed cellular immunity may also stay up to several months after surgery. For instance, a recent clinical study revealed that the decrease in total‐lymphocyte count and elevation of neutrophil‐to‐lymphocyte ratiopersisted for up to 6 months, in pancreatic adenocarcinoma patients who underwent curative pancreatectomy.^25^ The 6‐month postoperative period provides clinical physicians an immunological “window of opportunity” to kill cancer cells (Figure 1). Thus, in this review, we sought to outline the advances of surgical trauma and the subsequent biological responses contributing to immunosuppression. We will also summarize the possible therapeutic strategies based on surgical trauma‐related immunity dysfunction to reduce the postoperative tumor progression.

## 2 DAMPs, INFLAMMATION, NEUTROPHILS, AND MACROPHAGES IN SURGICAL TRAUMA‐INDUCED IMMUNOSUPPRESSION

**FIGURE 2:**
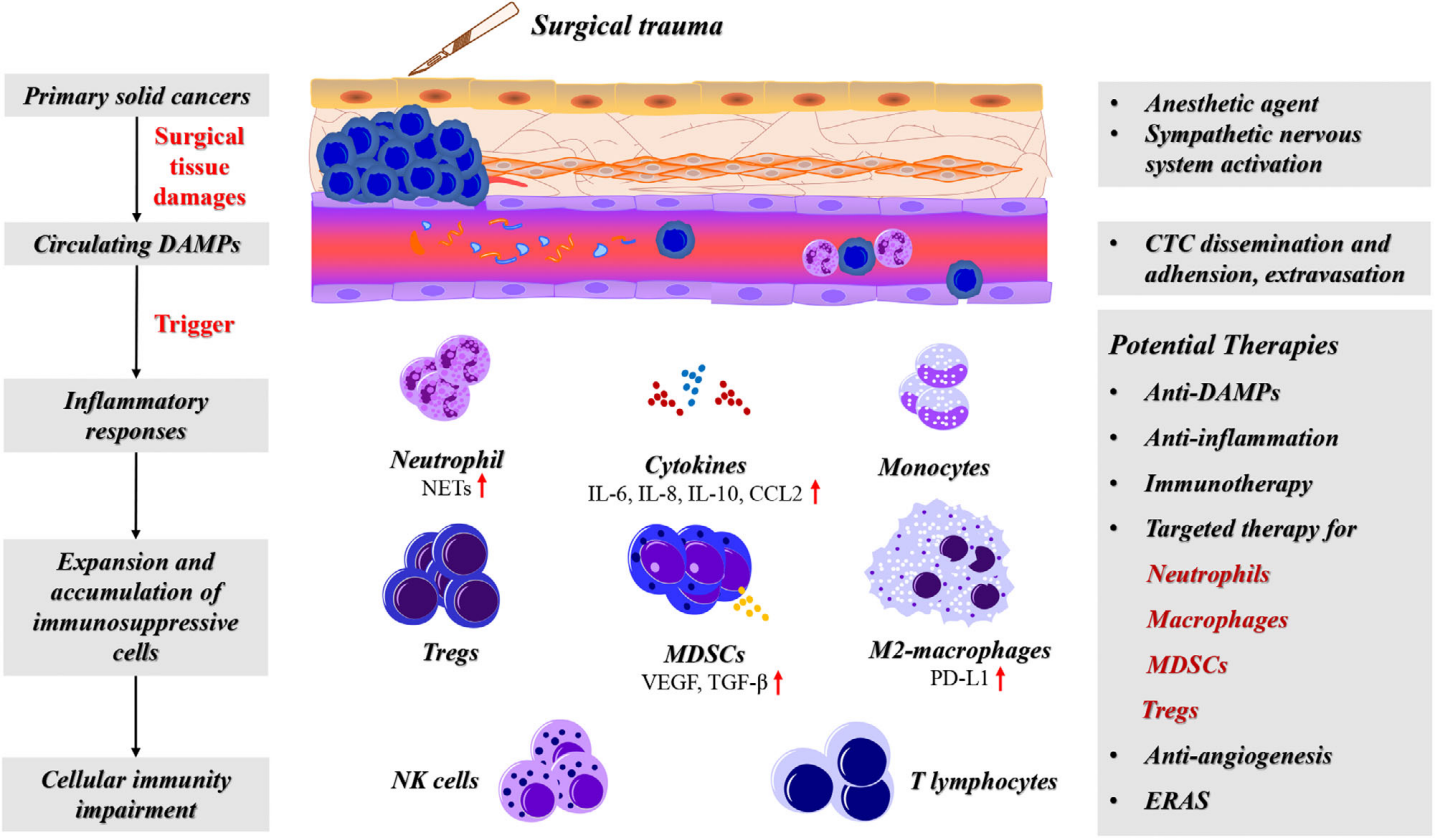
The schematic diagram of surgical trauma and the cellular immunity dysfunction. Tissue damages after surgery caused numerous DAMPs releasing into circulation. These DAMPs triggered a systemic and local organ inflammation response that disrupted the innate and adaptive immunity. Inflammatory metrics after surgery associated with the prognosis of cancer patients. The immunosuppressive cells including MDSCs, Tregs, and tumor‐associated macrophages are expanded under the surgical‐induced inflammation. Potential therapies to reduce the surgical trauma‐induced immunosuppression include anti‐DAMPs, anti‐inflammation therapies, combined immunotherapy with surgery, antiangiogenesis. Also, targeted therapies for MDSCs, Tregs, neutrophils, and macrophages may have surprising efficacy under the special condition in postoperative period. In addition, application of ERAS for patients with solid caners could accelerate the restoration of cellular immunity after surgery

Surgical trauma caused tissue damages that induced a large number of DAMPs released into circulation. These DAMPs initiated the innate immune defense and triggered a systemic and local organ inflammation. Under the inflammatory circumstances, the immunity dysfunction occurred. In addition, the activation of inflammasome and pyroptosis triggered by DAMPs produced chemokines to recruit the immunosuppressive cells included myeloid‐derived suppressor cells (MDSCs), M2‐macrophages, or regulatory T cells (Tregs) (Figure 2). In addition, the activated macrophage during DAMPs clearance or wound healing can be exploited by cancers for their metastasis.

### 2.1 DAMPs and the inflammatory pathways after surgical trauma

Following trauma, numerous mediators known as DAMPs are immediately released into circulation.^26^ The “alarmins” DAMPs may be nuclear or cytosolic proteins, extracellular matrix, or metabolic products^27^, ^28^, ^29^, ^30^, ^31^, ^32^, ^33^, ^34^, ^35^, ^36^, ^37^, ^38^, ^39^, ^40^, ^41^, ^42^, ^43^, ^44^ (Table 2). Plasma levels of DAPMs are associated with cellular immunity impairment in trauma patients.^45^ As intrinsic danger signals, DAMPs are sensitized and recognized via pattern recognition receptors (PRR) that mediated innate immune responses.^46^, ^47^ Toll‐like receptors (TLRs) are the main PRR for detection of DAMPs and TLR activation could predict the pathological conditions in trauma patients.^48^, ^49^ An important inflammatory signal TLR‐MyD88 was activated by DAPMs triggered a cascade signal response that increase the transcription activity of nuclear factor kappa‐B (NF‐κB).^44^

**TABLE 2:** The biological function of DAMPs released after solid cancer surgeries

Name	Category	Type	The PPR	Immunity‐related biological function	Reference
HMGB1	Proteins	Nonhistone chromatin nuclear peptide	TLRs 2, 4, 9 and RAGE	The increase in HMGB1 levels after surgery related with the decrease in HLA‐DR expression.	^27^
Interleukin‐1		Cytokines (IL‐1α and IL‐1β)	IL‐1R	Induce signaling cascades in target cells via MAPK or NF‐κB pathways.	^28^
Interleukin‐33		Nuclear alarmin	IL‐1RL1	Initiating the potential signaling pathway via NF‐κB and MyD88.	^29^
S100A proteins		Low molecular weight calcium‐binding homodimeric proteins	RAGE, TLR4	Activated p38 MAPK, ERK1/2, and transcription factor NF‐κB.	^30^
Histones		Epigenetic regulator	TLR2, TLR4 or TLR9	Extracellular histones induce multiple organ injury via direct endothelia disruption, and the subsequent release of other DAMPs.	31, 32
Complement factors		Effector arm of humoral immunity	Complement receptor	Activation of complement in trauma patients correlate with disease severity.	33, 34, 35
Heat shock proteins		Molecular chaperones	TLR2 and 4	HSP70 can stimulate monocytes/macrophages, and dendritic cells via TLR 2‐ and 4‐ pathways.	^27^
Nucleic acids	Nonproteins	Nuclear DNA, RNA	TLR3, TLR7, TLR8, TLR9, RAGE	Significantly increased in the immediate posttrauma period.	36, 37
Adenosine triphosphate		Metabolic DAMPs	Purinergic receptor P2 × 7	Contributing to the induction of inflammation by activation and recruitment macrophages, neutrophils, and dendritic cells.	^38^
Extracellular vesicles		Proteins, mRNAs, miRNAs, lipids	Cell‐to‐cell communicators	A significant increase in plasma extracellular vesicles after traumatic injury had proinflammatory effects that may influence outcomes.	^39^
Purine metabolites		Uric acid		Uric acid crystals act via inflammasomes, resulting in the production of active proinflammatory cytokines IL‐1β and IL‐18 and neutrophilic influx.	^40^
Biglycan and Hyaluronic acid		Extracellular matrix	TLR2, TLR4, NLRP3	Induce the secretion of TNF‐α, MIP‐1, and IL‐1β.	^41^
Succinate	Mitochondrial contents		Succinate receptor	Triggers pro inflammatory differentiation of lymphocytes.	^42^
Formyl peptides		Nucleoproteins	FP	Extracellular formyl peptides act as neutrophil attractants via FP receptors.	43
mtDNA			TLR9	Promote NLRP3 inflammasome activation, acute pulmonary inflammation, and injury through TLR9, p38 MAPK, and NF‐κB pathways.	44
TFAM		Mitochondrial transcription factor A	RAGE	Guides the TFAM‐mtDNA complexes to the endosomal pathways.	^42^

High mobility group Box 1 (HMGB1) and extracellular DNA are the two well‐studied DAMPs. HMGB1, a nonhistone chromatin nuclear peptide, acts as a DNA chaperon.^50^ The release of HMGB1 initiate an adaptive immune response that promotes malignant progression.^51^Trauma resulted in significant elevation in circulation HMGB1 as well as an increase of plasma‐activated TLR activation.^52^ Postoperative circulating DNA is associated with surgical invasiveness and was considered as a biomarker of the postoperative complications.^53^, ^54^ In severely injured patients, plasma mitochondrial DNA (mtDNA) DAMPs are associated with the evolution of system inflammatory reaction syndrome and mortality.^55^ In the tumor microenvironment, extracellular DNA promoted the cancer cells survival through induction of autophagy via TLR‐9 signaling.^56^

**FIGURE 3:**
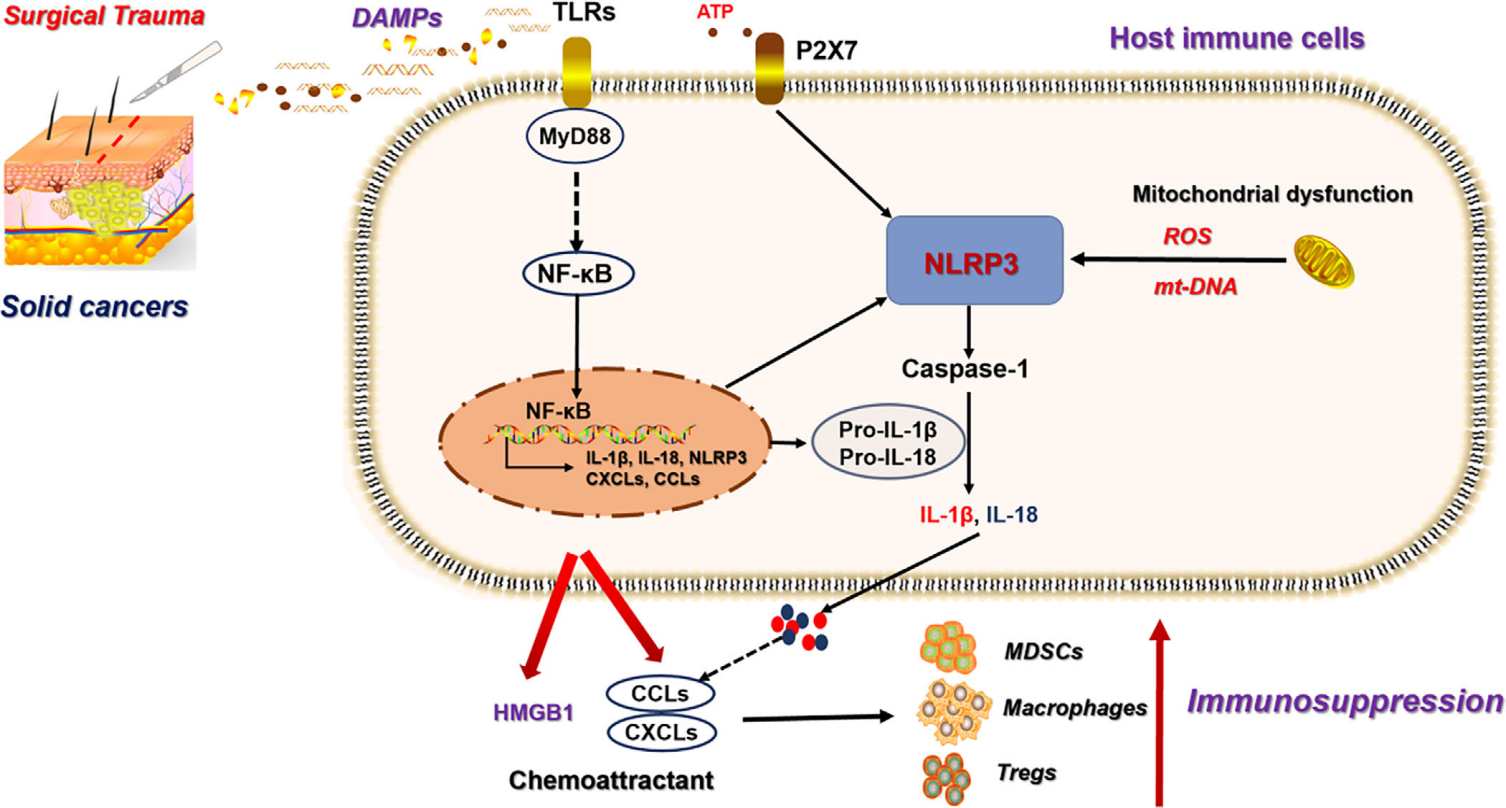
The mechanisms of surgical‐induced DAMPs and immunosuppressive cells accumulation. Surgical resection on solid cancers lead to tissue damages that releasing large amount of DAMPs into circulation. These DAPMs were recognized by PRRs on host immune cells that activate the NF‐κB transcriptional function. This process activates NLRP3 signal and the production of pro‐IL‐1β and pro‐IL‐18. Caspase‐1 cleaved the pro‐IL‐1β and pro‐IL‐18, increasing the secretion of IL‐1β and IL‐18, which promoted the secretion of CXCLs and CCLs. In addition, NF‐κB also upregulates the expression of CCLs and CXCLs expression, as well as the chemoattractant HMGB1

Some trauma‐induced DAMPs are also recognized by NOD‐like receptors (NLRs) that lead to an assembly of intracellular multiprotein complexes known as inflammasomes. NLRP3 inflammasome activation is one of the initial steps in an inflammatory cascade against DAMPs. NLRP3 activation promotes the translocation of the NF‐κB transcription factor to upregulate the expression of pro‐IL‐18 and pro‐IL‐1β. Caspase‐1 cleaved these frontiers, producing active IL‐1β and IL‐18. Through this process, inflammation was aggravated via recruitment of MDSCs, macrophages, and the promotion of pyroptosis (Figure 3). IL‐1β secretion is a marker of NLRP3 activation, which linked the tissue damages with tumor‐promoting inflammation.^57^ In breast cancer, fibroblasts sense DAMPs and activate the NLRP3 inflammasome, resulting in proinflammatory signaling upregulation and IL‐1β secretion. This signal facilitated tumor progression, which was attenuated when NLRP3 or IL‐1β was inhibited.^58^ Overall, the DAMPs released into circulation are considered main mediators of surgical trauma, and the subsequent inflammatory response, as well as immunity impairment, which is responsible for the outgrowth of circulating or dormant cancer cells.

### 2.2 Postoperative inflammation can predict the prognosis of solid cancers

Surgical interventions triggered a systemic and local organ inflammation that created an ideal environment for premetastatic niche formation and awaken dormant cancer cells. Open surgery, compared to laparoscopic surgery, has greater impact on inflammatory mediator production.^59^ Clinical data revealed that the inflammatory metrics like C‐reactive protein (CRP) and systemic inflammation score after surgery can be used to predict a poor clinical outcome in patients with solid cancers^60^, ^61^, ^62^, ^63^, ^64^, ^65^, ^66^, ^67^, ^68^, ^69^, ^70^, ^71^, ^72^, ^73^, ^74^ (Table 3). The negative correlation between postoperative inflammation and prognosis highlights the perspective efficacy of anti‐inflammation after surgery to improve the long‐term survival of solid cancers. Although inflammation is the normal defensive response to injury and infection, cancer cells hijack the process for their own survival.^75^ For example, one day after the injury, the inflammatory factors TNF‐α, IL‐6, and IL‐8 production capacity was significantly higher in patients who developed inflammatory complications compared with patients who did not, after a surgical intervention.^76^ In addition, the acute inflammatory response to surgical trauma favors the capture of tumor cells in foreign locations hence promoting metastasis. For example, proinflammatory cytokines such as IL‐1 and TNF‐α stimulated the adhesion of the circulating cancer cells.^77^ Under the inflammatory environment after surgery, dormant cancer cells can escape the surveillance of the immune system and manifest an outgrowth pattern. However, perioperative anti‐inflammatory treatment has been shown to significantly reduce the tumor outgrowth and restore the immunoresponses under such circumstances.^7^

**TABLE 3:** Recent clinical evidence about the correlation between postoperative inflammation and prognosis in solid cancers

Cancers	Surgery	Inflammatory index	Prognosis	References
Esophageal cancer	Transthoracic esophagectomy	C‐reactive protein (CRP)	Patients with intense postoperative inflammatory response showed a significantly shorter overall survival	^60^
Clear‐cell renal cell carcinoma	Nephrectomy	Systemic inflammation score (SIS)	A high SIS served as an independent prognostic factor of reduced overall survival	^61^
Colorectal cancer	Resection	Systemic inflammation score, postoperative Glasgow prognostic score (poGPS)	poGPS was associated with an incremental increase in the postoperative infective complication rates and a reduction in survival.	^63^
Colorectal cancer	Curative surgery	CRP	Complication severity, and postoperative day 4 CRP were associated with disease‐specific survival.	^63^
Lung cancer	Curative surgery	Complication and CRP	Postoperative white blood cell counts and CRP levels were significantly higher in those with postoperative respiratory complications than in those without. The incidence of postoperative respiratory complications was a significant predictor of cancer recurrence	^64^
Esophageal cancer	Radical esophagectomy	CRP	CRP value on postoperative day 4 may be useful for predicting serious infectious complications	^65^
Advanced gastric cancer	Curative resection	Hyperthermia and leukocytosis	Overall survival and relapse‐free survival were significantly worse in the prolonged hyperthermia group. The prolonged leukocytosis group showed significantly worse overall survival and relapse‐free survival	^66^
Locally recurrent rectal cancer	Radical surgery	Intraabdominal/pelvic inflammation	Intraabdominal/pelvic inflammation after radical surgery for locally recurrent rectal cancer is associated with poor prognosis	^67^
Colorectal cancer	Curative resection	Pre‐ and postoperative CRP	Combination of pre‐ and postoperative CRP levels was an independent prognostic indicator	^68^
Gastric adenocarcinoma	Curative surgery	Postoperative ratio (post‐CLR) of the maximum CRP value to the minimum peripheral lymphocyte count	Post‐CLR was an independent prognostic indicator for both the overall survival and disease‐specific survival	^69^
Lung adenocarcinoma or squamous cell carcinoma	Lobectomy	Postoperative blood monocyte count	Elevated early postoperative peripheral monocyte count was an independent prognostic factor of poor prognosis and inferior clinicopathological features	^70^
Resected colorectal cancer	Tumor resection	Postoperative netrophil‐to‐lymphocyet ratio (NLR), neutrophil, and monocyte to lymphocyte ratio (NMLR), platelet to lymphocyte ratio (PLR), and systemic immune inflammation index (SII)	Higher postoperative NLR, NMLR, PLR, and SII were associated with shorter progression‐free survival. Postoperative inflammation indexes and their dynamic changes, particularly for NMLR and SII are promising prognostic predictors	^71^
Stage I gastric cancer	Gastrectomy	CRP	The serum CRP level during the early postoperative period predicts the long‐term outcomes in stage I gastric cancer	^72^
Invasive bladder cancer	Radical cystectomy	NLR, monocyte‐to‐lymphocyte ratio (MLR), hemoglobin to platelet ratio (HPR) and CRP	A postoperative NLR at 3 months > 4.68 and a postoperative HPR at 3 months < 0.029 were associated with a significant reduction in cancer specific survival and overall survival	^73^
Thoracic esophageal squamous cell carcinoma (Stage I)	Subtotal esophagectomy	Maximum serum CRP level (CRPmax) and white blood cell count (WBCmax)	A high WBCmax in the early postoperative phase was an independent prognostic factor for poor overall survival	^74^

**TABLE 4:** The potential therapeutic strategies to overcome surgical trauma related cancer progression

Therapeutic strategies	Targets	Drugs	Mechanisms	References
Anti‐inflammation	NSAIDs	Aspirin, corticosteroids	Inhibit formation of fibrin and platelet clots	146, 147, 148, 149
	COXIBs	Celecoxib, Parecoxib	Exhibits relative selectivity for COX‐2 over COX‐1	145, 150, 151, 152
	IFN‐α	Infliximab	Activated the host immune response	^153^
	IL‐6	Siltuximab	Blocking the IL‐6–induced expression of proteins responsible for acute inflammation	^154^
DAMPs	Cell‐free DNA clearance	Nucleic acid scavenging microfiber meshes	Reduce the nuclei DNA induced inflammation	^129^
	Complement therapeutics	C1 esterase, C5a	Reduce tissue inflammation without blocking the complement cascade systemically	130, 131, 132
	HBMG‐1	Glycyrrhizin	Reverse and prevent activation of innate immunity and significantly attenuate damage in models of sterile‐induced threat	.^127^, ^128^
	TLR2	OPN‐305	Blocks the activation of TLR 2‐mediated innate immunity signaling	.^136^
	TLR4	MD2 inhibition (GLA‐SE)	Promotes strong Th1 and balanced IgG1/IgG2 responses to protein vaccine antigens	^137^
	TLR9	CpG‐C oligodeoxynucleotides	Senses CpG DNA in endosomes and induces the IFN response	^133^
		CMP‐001		^134^
	RAGE	FPS‐ZM1	Blocks the binding of amyloid β (Aβ) protein to RAGE and inhibits Aβ40‐ and Aβ42‐induced cellular stress in RAGE‐expressing cells	^135^
	NF‐κB	BAY11‐7082	Suppress NF‐κB activation and reducing the production of chemokines	^140^
	IL‐1β	Anakinra	Reduce the secretion of CCL2, CCL5, and CXCL5	^110^
	NLRP3	MCC950, CY‐09, OLT1177, Tranilast, and Oridonin	Directly target NLRP3 to downregulate the inflammatory and pyroptosis signal	^139^
Immunotherapy	Anti‐PD‐1	Pembrolizumab	Ameliorated T‐cell proliferation and partially reversed the T‐cell apoptosis induced by surgical trauma	124, 161
	Adoptive cell transfer	NKTT	Supplement the reduced number of NK cells after surgical stress	^160^
	Replicating viruses	Oncolytic viruses	Engage and mature conventional dendritic cells, which in turn activate NK‐ and T‐cells	^157^
	Prestimulation of immunity	Influenza vaccine	Administration 1 day before surgery, enhancing NK‐cell function through IFN‐α	158, 159
Neutrophil‐based therapy	Anti‐NET	DNAse I	Eliminating the NETs that format under surgical stress	^166^
	CXCR2	AZD5069, MK‐7123	Block the chemotaxis of neutrophil in acute inflammation	^164^
Macrophage‐based therapy	Minor groove of DNA Caspase 8	Trabectedin	Reducing the number of TAMs and the production of inflammatory cytokines and chemokines	182, 183, 184
	CSF‐1R	RG7155	Reduce the recruitment of macrophage and induced the apoptosis of activated macrophage	^171^
	TAM (TYRO3, AXL, MER)	RXDX‐106	Increased intratumoral CD8+ T cells and T cell function as indicated by both IFN‐γ production and LCK phosphorylation	^185^
	CD40	CP‐870,893	Reprogrammed TAMs create a proinflammatory environment that elicits effective T cell responses	172, 173, 174
	TLR9	IMO‐2055	Reprogramming protumoral macrophage to tumoricidal macrophage	175, 176, 177
	TLR7	Imiquimod	Phenotypic switch of TAMs to tumoricidal macrophages	178, 179, 180
	CD47	Hu5F9‐G4	A humanized, IgG4 isotype, CD47‐blocking monoclonal antibody, enables killing and phagocytosis of tumor cells by macrophages	^181^
	CCR2	PF04136309	Reduced the activated macrophage recruitment and regulated inflammation in wound healing	^170^
	CCL2	Carlumab	CCL2 increased after surgical wound, reduced the activated macrophage recruitment	167, 168, 169
MDSCs	Epigenetic therapy	5‐Azacytidine and entinostat	Downregulation of CCR2 and CXCR2 and promote MDSC differentiation into a more‐interstitial macrophage‐like phenotype	^186^
	PDE‐5	Sildenafil, Tadalafil	Downregulating ARG1 and nitric oxide expression	187, 188
	CXCR1/2	Reparixin, MK7123	Inhibit CXCR2^+^ G‐MDSC trafficking	190, 191
	ATRA	‐	Vitamin A derivative with antiproliferative properties	193, 194
	Vitamin D3	‐	Induce myeloid cell differentiation and enhance antitumor activity	^195^
	Gemcitabine/5‐FU	‐	Eliminate MDSC through induction of apoptosis	^196^
Treg based therapy	CD25	Daclizumab	Deplete CD4^+^CD25^+^ Treg cells and subsequently reduced Treg cell‐ mediated suppression of effector T cell function	206, 207
	CCR4	Mogamulizumab	Augmented the induction of cancer‐testis antigen (NY‐ ESO‐1)‐specific CD4^+^ and CD8^+^ T cells	208, 209, 210
	GITR	MEDI1873	Activation of antigen‐specific CD4+ effector T cells and selective depletion of Treg cells	^211^
	PI3Kδ	Parsaclisib	Treg cell maintenance and function are dependent on PI3Kδ signaling and inactivation of PI3Kδ in Treg cells resulted in increased activity of CD8^+^ T cells	212, 213
Antiangiogenesis	Monoantibody	Bevacizumab	A recombinant humanized monoclonal IgG1 antibody that binds to and inhibits the biologic activity of human VEGF	^204^
	Endostatin	Endostar	As postoperative complementary chemotherapy, due to the decrease of endostatin after surgery	^203^

### 2.3 Neutrophils are the first to respond surgical trauma induced‐inflammation

Being the most abundant white cells in circulation, neutrophils are the first to respond during inflammation.^78^ The roles of neutrophils in cancer immunotherapy and tumor progression have previously been implied. For instance, a high circulating neutrophil‐to‐lymphocyte ratio is a biomarker of poor clinical outcome in cancers.^79^ After abdomen surgery, the number of low‐density neutrophils with an immature phenotype is increased in peripheral blood in early postoperative period, which may promote the tumor recurrence in patients.^80^ Neutrophils undergo a process known as chemotaxis via the amoeboid movement, which enables them to migrate toward the sites of injury. Proinflammatory cytokines, including IL‐6, and LTB4, or CXCR2 chemoattractants pathways, are responsible for this biological behavior.^81^ Chemokine‐dependent neutrophil migration patterns result in enhanced tumor cell extravasation rates.^82^ In breast cancer, cell‐cell junction and cytokine‐receptor pairs define circulating tumor cells‐neutrophil clusters, highlighting key vulnerabilities of the metastatic process.^83^ Since the neutrophils aid movement and survival of the circulating cancer cells, inhibiting the proextravasation and prometastatic effect of inflamed neutrophils is necessary to restrict metastasis of the cancer.

Neutrophil extracellular traps (NETs) compromise a web of fibers composed of chromatin and serine proteases that trap and kill extracellular microbes.^84^ Surgical trauma induces the formation of NETs that promote tumor progression and lead to a poor prognosis. In a cohort of patients undergoing curative liver resection for metastatic colorectal cancer, an increase in postoperative NET formation was associated with a > fourfold reduction in disease‐free survival. Furthermore, an increase in NET formation correlated with an accelerated development and progression of metastatic disease, in a murine model of surgical stress.^85^ The metastatic dormancy is complicated in breast cancer, and NETs formatted after surgery have been shown to awaken the dormant cancer cells in mice under an inflammatory condition.^86^ Apart from awakening the dormant cancer cells, neutrophils also release NETs under the inflammatory condition which can capture circulating cancer cells via β1‐integrin‐mediated interactions, hence support the development of metastatic disease.^87^ Thus, treatments aimed to block the postoperative NET formation may benefit the patients undergoing resection of solid cancers.

**FIGURE 4:**
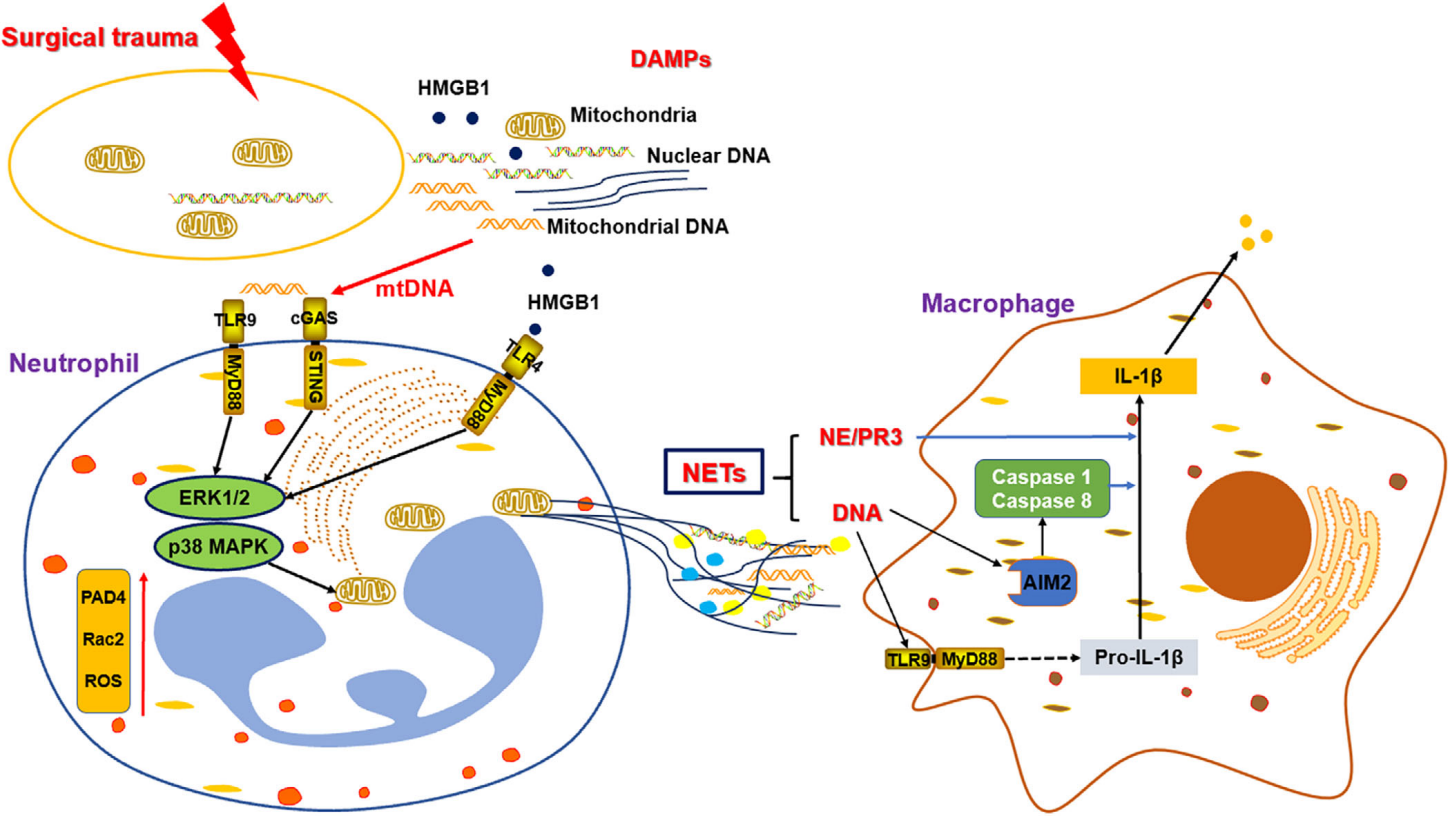
NETs formation induced by surgical trauma and the roles in immunosuppression. DAMPs releasing after surgical trauma included HMGB1 and mtDNA promote the NETs formation via TLR4‐ and TLR9‐MyD88, cGAS‐STING pathways. The main content of NET was mtDNA and could be regarded as DAMPs. NETs stimulate macrophages to secret chemoattractant via IL‐1β signals. NE: neutrophil elastase; PR3: Protease 3

DAMPs released after surgeries also induce the formation of NET via inflammatory signals. HMGB1 and histones induce NET formation via TLR4‐ and TLR9‐MyD88 signals. After depleting neutrophil, the adoptive transfer of TLR4 knockout or TLR9 knockout neutrophils conferred significant protection from liver ischemia/reperfusion injury with a significant decrease in NET formation.^88^ On the other hand, the components of NETs have been recognized as DAMPs, which trigger inflammatory signals to induce pyroptosis and inflammation. NETs induced macrophages producing IL‑1β via the caspase‑1 and caspase‑8 pathways (Figure 4). The secretion of IL‑1β upregulated the production of chemokines that recruit MDSCs or M2‐macrophages.^89^ Some studies have revealed that the main content of NETs formed after surgery are mtDNA, with no detectable nuclear DNA component.^90^ In an acute peripheral tissue trauma model, the release of mtDNA induced the formation of NETs and sterile inflammation via cGAS‐STING and the TLR9 pathways.^91^ These results indicate that clearance of the postoperative DAMPs and blockade of the related inflammatory pathways could reduce the formation of NETs, thus decreasing the early recurrence or metastasis of cancers.

### 2.4 Cancers exploit the activated macrophages during wound healing for their metastasis

Cancer has often been described as a “wound that does not heal,”^92^ since the two share some common features. In melanoma, a strong correlation exists between neutrophil at sites of melanoma ulceration and cell proliferation, which is associated with poor prognosis.^93^ Most of the growth factors, chemokines, and cytokines detected during wound healing promote tumor growth, invasion, or angiogenesis. For example, factors such as transforming growth factor‐α (TGF‐α) and transforming growth factor‐β (TGF‐β), insulin‐like growth factor I and II (IGF I and II), and platelet‐derived growth factor have been proved to play a role in the different stages of wound healing. Some of these factors were involved in the outgrowth of tumors.^94^ In metastatic mouse breast cancer‐bearing mice, injection of wound fluid close to the tumor site containing cytokines or growth factors accelerated the tumor growth.^95^, ^96^ Wounding after surgery increased ovarian tumor mass and decreased perioperative cisplatin efficacy in a separate study.^97^ Besides promoting the growth of cancer cells, most of the signaling molecules and cells involved in wound healing have also play a role in escape from tumor dormancy and the development of metastases.^98^

Macrophages circulate as monocytes, until activation when they migrate to the injury site and become macrophage.^99^ The first responder neutrophils dominate the early stages of inflammation setting the stage for the repair of tissue damages by recruited macrophages.^100^ HMGB1 induced hepatocytes to produce PC3‐secreted microprotein, which is a potentially attractive therapeutic target for decreasing macrophage infiltration after surgery.^101^ The phenotypes of wound‐healing macrophages tend to be similar with tumor‐associated macrophages that be exploited by cancer cells to their advantage. For example, tumor‐associated macrophages expressing fibroblast activation protein alpha and hemoxygenase‐1 have similar phenotype to that observed during the wound‐healing response. The expression of a wound‐like cytokine response within the tumor is clinically associated with poor prognosis in certain cancers. Some tumors exploited this response to facilitate metastasis.^102^ However, the macrophage phenotype from animal models cannot represent the characteristic of human macrophages. In a clinical study on human wound‐healing response, M‐1 macrophages had perivascular distribution, while M‐2 macrophages were diffusely distributed in the dermis. In addition, M‐1 macrophages were the main source of TNF‐α and IL‐6 in wound, while arginine (ARG1) correlated with M‐2 macrophage density.^103^ Even though these evidences exist, completely deleting macrophages to prevent cancer progression remains a controversial subject. A balance should however be struck between wound healing and how to limit cancer cells from taking advantage of these immune cells.

## 3 THE EXPANSION OF MDSCS, Tregs, AND ELEVATED PD‐L1 EXPRESSION AFTER SURGERY

### 3.1 Surgical trauma enhances the expansion and recruitment of MDSCs

MDSCs promote tumor metastasis by inducing the formation of a premetastatic niche, strengthening the angiogenesis and tumor cell invasion. However, surgical stress triggers several changes in phenotype and function, including enhanced the expansion and recruitment of MDSCs. Besides revealing that an increased frequency of postoperative CD14^+^HLA‐DR^−/low^ MDSC was correlated with early recurrence, a clinical study also predicted the prognosis of patients with hepatocellular carcinoma patients undergoing curative resection.^104^ Vascular endothelial growth factor (VEGF) is an important cytokine secreted by MDSCs that contribute to the angiogenesis, and its concentration in serum of lung cancer is often elevated. Such increased concentration has been correlated with the concentration of MDSCs 1 week after surgery.^19^ The elevation of the circulating VEGF level after surgery implies that antiangiogenesis can be used to inhibit the outgrowth of cancer metastasis.

Following the MDSCs expansion and recruitment after surgery, the function of NK cells which is critical for killing the circulating cancer cells is impaired. Therefore, perioperative treatments aimed at enhancing the function of NK cells could lower the metastatic recurrence and improve the survival of surgical cancer patients.^105^ In addition, the impairment of T cells was also associated with the expansion of MDSCs after surgery. Besides, in a mouse model of cancer resection, MDSC population numbers and functional impairment of tumor‐associated antigen‐specific CD8^+^ T‐cells were altered under surgical stress.^106^ Although arginine is an essential amino acid for normal T‐cell function, its availability decreases after a physical injury, which may inhibit the function of T‐cells after injury.^107^

Besides promoting the expansion of MDSCs, proinflammatory cytokines released after tissue damages including IL‐6, TGF‐β, and IL‐10 activate inflammatory signaling cascades including MyD88 and the transcriptional factor NF‐κB.^108^, ^109^ The following activated IL‐1β associated with increased secretion of CCLs or CXCLs that responsible for monocytic‐MDSCs (M‐MDSCs) and granulocytic‐MDSCs (G‐MDSC) accumulation, respectively.^110^ In addition, prostaglandin E2 (PGE‐2)^111^, ^112^ and TNF signal^113^ also involved in the process of MDSCs accumulation. However, in colon cancer, surgical trauma promoted tumor progression by downregulating CXCL4 and inducing the MDSC recruitment, which lead to immunity‐suppressive environment.^114^

Some DAMPs can function as chemoattractant that directly recruit MDSCs. Expression of S100A8 in the lungs promotes MDSC accumulation, which suppresses a localized immune response.^115^ DAMPs such as S100A8 and S100A9, released after surgery, are potent chemoattractants for MDSC.^116^ S100A8/A9 induced serum amyloid A3 that directly recruited MDSC in lungs and facilitated metastasis.^117^ HMGB1 was released in large amounts after abdominal surgery, which recruits numerous MDSCs to promote peritoneal metastasis of colon cancer.^118^ In addition, HMGB1 contributed to tumor progression though increasing the viability of MDSCs by driving them into a proautophagic state.^119^ These evidences show that the mechanisms of the MDSCs expansion under surgical stress may be complex. Therefore, phenotyping the recruited MDSCs responsible for premetastatic niche formation after surgery could provide insights for the related targeted therapy.

### 3.2 The number of Tregs increases under surgical stress

Treg is a highly immunosuppressive CD4^+^ T cell that migrate into inflammatory sites and suppress effector lymphocytes such as CD4^+^ T helper cells and CD8^+^ T lymphocytes.^120^ Tregs serve to maintain the immune homeostasis, which get expanded under surgical stress. Surgical trauma promoted colon cancer progression by increasing CCL18 expression thereby promoting the recruitment of Treg.^121^ In patients who received a radical mastectomy, the peripheral Treg levels were markedly elevated on the seventh day postoperation.^13^ After gastric surgery, the percentage of Treg cells increased, accompanied by elevated expression of Foxp3 and TGF‐β1, and decreased T helper cells. In addition, the expression of programmed death‐1 (PD‐1) and programmed death ligand‐1 (PD‐L1) increased and the polarization of Tregs was only inhibited after anti‐PD‐1 treatment.^122^ Since patients with early stage I/II tumors showed decrease Treg population, while those with late stage III/IV tumors presented greater amounts,^123^ accumulation of Treg cells after surgery may be dependent on tumor stage. Tregs exert their immunosuppressive activity through various cellular and humoral mechanisms. Therefore, identifying the immunity phenotype of Treg expanded under the surgical stress and developing the related targeted therapies may have promising prognosis under the special condition after surgery.

### 3.3 Expression of PD‐L1 increases under surgical stress

Surgical trauma increased PD‐1/PD‐L1 expression on immune cells. In in vivo studies, administration of anti‐PD‐1 antibody significantly ameliorated T‐cell proliferation and partially reversed the T‐cell apoptosis induced by surgical trauma.^124^ Besides, in a surgical stress mice model, surgical stress reduced the total number of CD8^+^ T cells and impaired the function of cytotoxic lymphocytes. Conversely, blocking PD‐1 with specific antibody restored the numbers and the secretion ability of CD8^+^ T cell. In addition, expression of PGE2 was dramatically upregulated, and anti‐PD‐1 plus PGE2 inhibitor restored cytotoxic T lymphocytes dysfunction after surgery.^125^ Under systemic inflammation induced by surgical stress, the expression of PD‐L1 on activated macrophages outnumber carcinoma cells, suggesting a prominent role for tumor‐associated macrophage‐associated PD‐L1 in immunosuppression and tumor outgrowth.^7^ For instance, gastric cancer resection altered the balance of Th17/Treg cells and increased PD‐1/PD‐L1 expression, while transfection of Ad‐sh‐PD1 in an in vitro study ameliorated Th17/Treg cell imbalance partially by increasing the expression of miR‐21.^126^ These lines of evidence suggest that the combination of surgery and immune checkpoint inhibitor may have a synergy that can be exploited to reduce tumor progression after surgery.

## 4 POTENTIAL THERAPEUTIC STRATEGIES

The postoperative period provides a special immunological opportunity for cancer therapy. Compelling clinical evidence show that postoperative inflammation is common among solid cancer patients. Acute inflammation after surgery is a kind of innate response, and the alterations of immunity in this period suggest that the current therapies which are ineffective in normal conditions may actually be effective during this period after surgery (see Table 4). In addition, treatment options should balance the acute surgery‐induced inflammation, wounding healing, and cancer metastasis. Solid cancers with a large tumor burden may have a relative abundance of circulating tumor cells, or micrometastatic lesions and will take advantage of this special postoperative period for their outgrowth. Many chemokines, growth factors, cytokines and immunity cells like neutrophils and macrophages are also involved in the acute inflammatory response after surgery and during wound healing. Under this condition, some immunosuppressive cells like MDSCs and Tregs are activated and amplificated, then migrate to targeted organs, resulting in the formation of a premetastatic niche. Deletion of neutrophils and macrophages after surgery may however not be the best way to reduce the postoperative cancer progression since these cells are essential for normal immune response to injury. The immune homeostasis limits the period of immunodysfunction within 2 weeks after surgery, even though the impact on the immunity or survival of patients may be longer. Therapeutic strategies aimed at targeting DAMPs released after surgical trauma, anti‐inflammation, clearance of NETs, preventing the activated macrophages migrating to distant organs, and targeted therapy for MDSCs, or Tregs, may have superior efficacy.

### 4.1 Anti‐DAMPs and the related inflammatory signals

Anti‐DAMPs and the related inflammatory/pyroptosis signal are the overall strategy based on surgical trauma to reduce the postoperative immunosuppression. Strategies for targeted DAMPs therapy included specific DAMP inhibition, blockage of the related PRR pathways such as TLRs or receptor of advanced glycation endproducts (RAGE), and blockage of the NF‐κB, NLRP3, and IL‐1β signal. HMGB1 is involved in many aspects of trauma‐induced immune suppression. Treatment using anti‐HMGB1 monoclonal antibody antibodies was found to ameliorated impairment of the trauma‐induced T cells activity and accumulation of MDSCs, 2 days after injury.^127^ In addition, the postoperative immunosuppression was also associated with infectious conditions like sepsis. Anti‐HMGB1 antibodies were reported to attenuate inflammation in murine sepsis models and reduce mortality without potentiating immunosuppression.^128^ Clearance of cell‐free DNA improved the clinical outcome in some inflammatory diseases. In fact, nucleic acid scavenging microfiber meshes represent an effective strategy for combating inflammation and thrombosis in trauma.^129^ Other therapeutic options included anticomplement system therapies^130^, ^131^, ^132^ and drugs against PRR receptors. For instance, administration of the TLR9 inhibitor ODN2088 was found to inhibit mtDNA‐triggered systemic inflammation.^133^ Similarly, CMP‐001, a virus‐like particle that encapsulating an immunostimulatory CpG‐A oligodeoxynucleotide TLR9 agonist, was reported to CMP‐001 induced production of cytokine IFN‐α from plasmacytoid dendritic cells.^134^ In addition, RAGE inhibitors, such as FPS‐ZM1, a novel class of RAGE inhibitors screened from 5000 small molecules, have been reported to inhibit cancer progression and metastasis.^135^ Furthermore, OPN‐305, a first humanized IgG4 monoclonal antibody against TLR2, was reported to produce full TLR2 receptor blockade on monocytes, with a linear effect on the inhibition of IL‐6 release after TLR2 stimulation.^136^ Other inhibition strategies have employed MD2 inhibitor (GLA‐SE) with adjuvant GLA‐SE found to promote expansion in human T follicular helper cells and the emergence of public TCR‐β clonotypes.^137^

As the secretion of chemokines related to activated inflammatory or pyroptosis pathways, blockage of NF‐κB, IL‐1β, and NLRP3 is presented as promising therapeutic strategies to reduce the accumulation of postoperative immunosuppressive. In mouse breast cancer, blocking IL‐1β reversed the immunosuppression and synergized with anti‐PD‐1 for tumor abrogation.^138^ Anakinra is an IL‐1 receptor antagonist with the potential to inhibit distal metastasis via pyroptosis/IL‐1β pathways blockade. Administration of anakinra reduced the recruitment of M2‐macrohages and MDSCs via downregulation of CCL2, CCL5, and CXCL5 secretion.^110^ Inhibitors specific to NLRP3 inflammasome may be the best option for therapy of NLRP3‐related disease. However, even many small molecules have been identified as NLPR3 inflammasome inhibitors, few of them have been clinically studied for human diseases. Among them, MCC950, CY‐09, OLT1177, Tranilast, and Oridonin displayed the good therapeutic properties, as they directly target NLRP3.^139^ BAY11‐7082 is a NF‐κB inhibitor and reduced the secretion of CXCL1, CXCL2, and CXCL8 which are essential chemokines for CXCR2^+^ MDSCs.^140^ However, most of the aforementioned drugs have not been used at the postoperative period. In this work, these anti‐DAMPs treatments are presented to highlight their potential and promise as therapies for improving clinical outcomes of patients with solid cancers after surgery.

### 4.2 Anti‐inflammation therapies

Nonsteroidal anti‐inflammatory drugs (NSAIDs) effectively inhibited systemic inflammation and eliminated suppression of NK cell populations after surgery.^141^, ^142^ Aspirin presents anticancer properties by suppressing inflammatory cytokines that regulate cell proliferation, angiogenesis, and apoptosis. In patients with bladder cancer, daily aspirin administration was associated with significantly improved survival after radical cystectomy.^143^ With a combination therapy comprising aspirin and NSAIDs, after surgery, lowered the risk of early recurrence of breast cancer, and colorectal cancers as well as hepatocellular carcinoma.^144^, ^145^, ^146^ Dexamethasone, a classic anti‐inflammation option and a single intraoperative dose of dexamethasone, was independently associated with improved overall survival rates in patients with pancreatic adenocarcinoma.^147^ However, the efficacy of anti‐inflammation therapies in inhibition of postoperative tumor progression remains unclear. For example, Elecoxib, a COX2‐specific inhibitor, did not significantly affect apoptosis in prostate, and breast cancer as well as cervical intraepithelial neoplasia.^142^, ^148^, ^149^ Similarly, intraoperative use of a single dose of parecoxib, another NSAIDs drug, was not associated with decreased cancer recurrence after bladder cancer surgery.^150^

As the anti‐inflammatory agents also directly inhibit tumor growth,^151^, ^152^ in order to explore the efficacy of these drugs that have not been inferred, in a wounded‐mouse prior to breast cancer cells injection that aimed to eliminate the influence of the primary tumor, treatment with meloxicam altered the phenotype of tumor‐associated macrophages. Before meloxicam administration, distant surgical wounding induced upregulation of CD206 on the surface of tumor‐associated macrophages, indicative of a protumor M‐2 polarization that is often implicated in immunosuppressive properties. Administration of wounded mice with meloxicam prevented the increased CD206 expression and led to a decrease in PD‐L1 expression on tumor‐associated macrophages.^7^ This result demonstrated the influence of surgical trauma‐induced inflammation on the immunosuppression phenotype of innate immune cells. A combination treatment compromising inhibition the COX and blockage of PD‐1 indicates a synergistic efficacy in inducing eradication of tumors.^152^ Inhibition of other proinflammatory factors, like IL‐6, and IFN‐α, in some inflammatory diseases has also been documented under normal conditions.^153^, ^154^ It is possible that these may have varied efficacies under postoperative condition.

### 4.3 Combination therapy of immunotherapy and surgery

Combining perioperative immunotherapy with standard‐of‐care surgery has the potential to improve survival in surgical cancer patients.^155^ Surgical attack causes a global dysfunction in NK cells. However, perioperative therapies aimed at enhancing NK cell function could reduce metastatic and recurrent rates in patients received solid cancer resection.^156^ For example, preoperative administration of replicating viruses, such as novel anticancer oncolytic viruses, was reported to effectively prevent postoperative NK‐cell dysfunction.^157^ Similarly, stimulating the immune system using nonreplicating viral vaccines, such as influenza vaccine, prevented postoperative metastases by enhancing NK‐cell function through IFN‐α. However, this strategy requires an accurate timing and is therefore applied 1 day before surgery, to allow for sufficient time for optimal activation of NK cells prior to surgical stress.^158^, ^159^ NK cell–based transfer therapy is another attractive method for overcoming NK cells dysfunction after surgical stress. However, this strategy has not been tested in the perioperative period, due to the highly cytotoxic nature of NK cells.^160^

Upregulation of PD‐L1 expression in immunosuppressive macrophages after surgical stress indicates that blockage of PD‐1 using monoclonal antibodies may be an effective treatment therapy for restoring immunosuppression. In a surgical stress mice model, blockage of PD‐1 using specific antibodies restored CD8^+^ T cell numbers as well as their secretion ability. In addition, PGE2 expression was dramatically upregulated after surgery, and anti‐PD‐1 plus PGE2 inhibitors restored the dysfunction of cytotoxic T lymphocytes induced by surgery.^161^ Herpes virus entry mediator (HVEM) is a new potential mediator of trauma‐induced immunosuppression, with surgical trauma patients exhibiting greater expression levels of HVEM^+^/CD3^+^ lymphocytes.^162^ The identification of novel surface markers on effective lymphocytes, after surgery, could contribute to development of related immunotherapies.

### 4.4 Targeted therapies for neutrophils and NETs

Neutrophils function as the main innate defense factors during early stages of injury. Apoptosis of neutrophils is programed cell death and helps to maintain immune homeostasis. However, inflammatory responses due to tissue injury disrupts programmed neutrophil death, leading to a dysfunction of immunity. Doxorubicin‐conjugated protein nanoparticles can be used for selective in situ targeting of inflammatory neutrophils for intracellular delivery of doxorubicin to induce apoptosis.^163^ In addition, the CXCR2 signaling pathway is a potential target for modifying neutrophil dynamics in inflammatory diseases. For instance, AZD5069, a CXCR2 antagonist, was reported to selectively reduce absolute neutrophil counts in asthma patients.^164^ This treatment may also reduce the infiltration of these inflammatory neutrophils to the premetastatic organ. However, AZD5069's potential as a therapeutic agent for cancer patients is still under investigation. Another CXCR2 antagonist, MK‐7123, is under evaluation with pembrolizumab in patients with selected advanced/metastatic solid tumors (NCT03473925).

Studies targeting neutrophils‐releasing neutrophil elastase in cancer patients have been reported. For instance, administration of sivelestat sodium hydrate, a selective inhibitor of neutrophil elastase after transthoracic esophagectomy was found to improve the condition of systemic inflammation and postoperative clinical courses.^165^ There is no direct report about elimination of NETs by DNase I digestion to minimize the postoperative tumor progression. Although several clinical trials are ongoing, no results have been reported regarding targeting NET in patients with cancers.^166^ Anti‐NET may have promising clinical benefits for patients under the specific conditions, including the postoperative period.

### 4.5 Targeted therapies for macrophages

Current strategies focusing on macrophage‐targeted therapy include blocking macrophage recruitment, inducing apoptosis of tumor‐associated macrophages, and their reprogramming to release cancer promotion phenotype. Recruitment of macrophages or inflammatory monocytes mostly depend on the CCL2‐CCR2 chemokine axis. Levels of circulating CCL2 increased immediately after surgical trauma, indicating its potential efficacy in targeted therapy. Carlumab, a human anti‐CCL2 IgG1κ mAb, presents preliminary antitumor activity.^167^, ^168^, ^169^ Similarly, a combination therapy, comprising PF‐04136309, a CCR2 inhibitor with FOLFIRINOX, achieved generated effective tumor response and achieved local tumor control in 97% of patients with pancreatic cancer.^170^ In addition, blockade of the IL‐1β/pyroptosis pathway by the IL‐1 receptor inhibitor anakinra reduced the secretion of CCL2 and the following M2‐macrophage recruitment.^110^ CSF‐1R binds to CSF1 and IL‑34 to regulate macrophage differentiation, proliferation, and survival. Administration of Emactuzumab, a humanized mAb that binds to CSF‐1R and blocks its dimerization, was found to reduce recruitment and induce apoptosis of tumor‐associated macrophages. A combination therapy involving CSF‐1R and Emactuzumab has also been reported. For instance, combining CSF‐1R inhibition with Emactuzumab in locally advanced diffuse‐type tenosynovial giant cell tumors achieved a response in 24 (86%) out of 28 patients, with two (7%) exhibiting a complete response.^171^ Another promising strategy involves rebalancing microenvironment immune infiltration from a protumoral status to effective antitumor in synergy with T cell‐enhancing drugs. Current therapies for reprograming macrophages include the use of anti‑CD40, anti‑CD47, and antibodies as well as TLR agonists.^172^, ^173^, ^174^, ^175^, ^176^, ^177^, ^178^, ^179^, ^180^, ^181^

Trabectedin was reported to reduce the number of tumor‐associated macrophages via G2‐M phase cell cycle arrest and apoptosis.^182^ In addition, Trabectedin monotherapy reduced the risk of disease progression in patients with advanced translocation‐related sarcoma.^183^, ^184^ Pan‐tumor association macrophages small‐molecule kinase inhibitor RXDX‐106 (TYRO3, AXL, MER) activates both innate and adaptive immunity to inhibit the growth and progression of tumors. In addition, RXDX‐106 also potentiated the effects of α‐PD‐1 Ab, leading to enhanced antitumor efficacy and survival.^185^ A better understanding of the mechanisms underlying how cancers utilize activated macrophages during wound healing is needed. Preventing activated macrophages from travelling to distant organs and promoting the formation of premetastatic niche, after surgical trauma, would be a key step in the targeted therapies. Furthermore, identifying ideal markers for targeted therapy, as well as phenotyping the macrophages during wound healing are also critical in development of targeted therapies for macrophages.

### 4.6 Targeted therapies for MDSCs

To date, no agents that specifically target MDSC have been developed, due to the heterogeneous features and a lack of ideal markers for selective targeting. However, there were still some drugs that target MDSC have been developed and are indirectly used for by cancer patients. A recent study revealed that administration of low‐dose DNA methyltransferase and histone deacetylase inhibitors inhibited the trafficking of MDSCs through the downregulation of CCR2 and CXCR2 and promoted MDSC differentiation into a more‐interstitial macrophage‐like phenotype.^186^ A preclinical study reported that perioperative phosphodiesterase‐5 inhibition with sildenafil reduced the function of surgery‐derived G‐MDSC. In addition, sildenafil plus perioperative influenza vaccination removed surgery‐derived immunosuppressive mechanisms of MDSCs and reduce postoperative metastasis.^187^, ^188^ Studies have implicated C‐X‐C motif chemokine pathways in the recruitment of G‐MDSCs. Here, tumor cells secrete CXCL1 via the VEGF pathway that interact with the CXCR2 pathway to recruit G‐MDSCs. Administration of and a combination of Reparixin, an inhibitor of CXCR1/2, with weekly paclitaxel in patients with HER‐2‐negative metastatic breast cancer achieved a 30% response rate.^189^ Administration of this drug also attenuated postoperative granulocytosis in patients who received on‐pump coronary artery bypass graft surgery.^190^ In addition, administering MK‐7123, a specific CXCR2 inhibitor reduced neutrophil chemotaxis and alleviated airway inflammation in chronic obstructive pulmonary disease (NCT 01006616).^191^ Furthermore, other potential therapeutic targets are currently under preclinical evaluation. Snail involving the CXCR2 chemotaxis of MDSCs and snail‐knockdown reduced the expression of CXCR2 ligands, CXCL1, and CXCL2.^192^ Other drugs that inhibition of MDSCs in cancer patients included all‐trans retinoic acid,^193^, ^194^ vitamin D3,^195^ and Gemcitabine/5‐FU.^196^ The expansion and recruitment of MDSCs after surgical trauma forms a premetastatic niche in distant organs that favor the colonization of circulating cancer cells. Preventing expansion of MDSCs after surgery is a much rational strategy to alter the postoperative “fertile soil” for metastasis, with low risk of wound‐healing problems. Even though limited drugs have been used under this condition, future studies are expected to explore the relevant therapies directly used after surgical trauma. Furthermore, it will be important to understand the mechanisms underlying expansion and recruitment of MDSCs after surgery.

### 4.7 Antiangiogenesis after surgery

Studies have reported an increase in serum VEGF increased after solid tumor resection, indicating that antiangiogenesis is a promising option to slow postoperative tumor progression.^197^, ^198^, ^199^ Functionally, expanded and recruited MDSCs in premetastatic organs after surgery secrete the angiogenetic factor VEGF.^200^ Antiangiogenesis drugs have achieved significantly favorable efficacy in some solid cancers, suggesting the potential of these drugs in prevention of postoperative angiogenesis. In patients with colorectal cancer undergoing radical surgery, IL‐2 preoperative immunotherapy partially reduced VEGF increase during the postoperative period. This evidence suggested that presurgical immunotherapy using IL‐2 may counteract surgery‐induced stimulation of the angiogenesis by reducing the increase of the angiogenic factor VEGF.^201^ Moreover, Endostatin was found to significantly decrease after primary osteosarcoma removal, while treatment with the antiangiogenic reagent TNP‐470 suppressed postoperative progression of pulmonary metastasis in osteosarcoma.^202^ In patients with nonsmall cell lung cancer, postoperative complementary chemotherapy comprising application of recombinant human endostatin (Endostar) achieved a significantly increased average progression‐free survival in a treatment group by 9.8 months, which was significantly different compared to the control group.^203^ Bevacizumab is an antineoplastic agent, which acts to prevent angiogenesis thereby preventing or reducing metastatic progression. Perioperative bevacizumab benefits patients who have undergone lung metastasectomy.^204^ However, when administered in the perioperative phase in patients with solid cancers, antiangiogenic agents may have drawbacks on biological activity that involve physiological angiogenesis, such as wound healing. Such treatment should, however, be administered with caution in the perioperative period until the therapy is validated using results from large clinical trials.^205^

### 4.8 Targeted therapies for Tregs

Strategies for targeting Tregs included targeting the receptors on Tregs or the intracellular signal. Consequently, studies have focused on evaluating the effects of depletion of Tregs by targeting CD25 with antibodies or IL‐2R with a recombinant protein composed of IL‐2.^206^, ^207^ CCR4^+^ Tregs with strong immunosuppressive activity is present in the blood of patients with melanoma. Mogamulizumab, an anti‐CCR4 antibody, is currently being explored for its efficacy in patients with advanced‐stage solid tumors (NCT02281409 and NCT01929486).^208^, ^209^, ^210^ In addition, MEDI1873, a novel, potent GITR agonist has the ability to regulate T‐cell responses.^211^ This GITR agonist, individually or in combinations, is currently being investigated for its efficacy in advanced stage solid cancers (NCT02583165 and NCT02628574). In addition to the aforementioned compounds, signals crucial for Tregs survival and functions are also promising targets for Tregs‐related therapies. For instance, a combination treatment of Parsaclisib, a selective phosphatidylinositol 3‐kinase δ (PI3Kδ) inhibitor, is currently being explored during the initial stages of a phase I trial in advanced solid cancers.^212^, ^213^

### 4.9 Enhanced recovery after surgery

Surgical stress influences oncological outcomes and survival of cancer patients. Consequently, an enhanced recovery after surgery (ERAS) protocol designed to reduce perioperative stress has subsequently shown potential in reducing postoperative inflammation as well as the related morbidity. In patients with nonmetastatic colorectal cancer undergoing laparoscopic surgery, the ERAS protocol decreases risk of death at 3 years after surgery by 56% in patients compared to the control group.^214^ In gastric carcinoma patients, ERAS reduced postoperative inflammation and improved cellular immunity. In addition, patients subjected to ERAS exhibited higher serum albumin and prealbumin levels at day 7 postoperation. With regard to cellular immunity, the total T lymphocytes were higher in patients on whom ERAS was performed on postoperative day 3.^215^ Despite these benefits of ERAS on short‐term results in cancer patients, little is known about its impact on long‐term results. In patients with colorectal cancer, application of the ERAS protocol is associated with improved 5‐year survival after surgery, with low CRP levels on postoperative day 1.^216^ Therefore, the ERAS protocol improves the postoperative status and quick recovery of the immunity, benefiting patients with solid cancers under severe surgical stress.

## 5 CONCLUSION AND FUTURE PROSPECTS

Surgical resection remains the main treatment for localized solid cancers. The immediate postoperative period indeed supplies an immunological “window of opportunity” for developing relevant therapies to eliminate the circulating cancer cells or the dormant cancer cells. To achieve this, understanding the postoperative biological changes is critical. Previous studies have described the impact of anesthesia, the alteration of neuroendocrine, circulation system, and sympathetic nervous systems on postoperative cancer progression. Clinical evidence shows that immunosuppressive status correlates with the severity of surgical trauma, while postoperative inflammation is a predictor for the prognosis of cancer patients. Altering inflammation alteration after surgery and the significance of clinical relevance put the tissue damages after surgical manipulation at the center of the postoperative immunosuppression. Surgical patients exhibit high levels of circulating DAMPs, which trigger the local and systemic inflammation, associated with immunity dysfunction. Cancer cells take advantage of the activated or expanded effector immunosuppressive cells such as MDSCs, Tregs, macrophages, and neutrophils to favor their progression. However, the exact mechanisms underlying the immunological alteration of these cells as well as their phenotype remain unclear. Since the related targeted therapies have developed very slowly, it is imperative to evaluate effective therapies for application under special condition during the postoperative period. Most of the anti‐inflammatory drugs have improved the survival of patients after cancer resection. However, the timing for their administration is critical. Other therapies including immunotherapy, targeted therapies based on immunosuppressive cells, antiangiogenesis, as well as enhanced recovery protocol are also effective. Preoperative stimulation of the immune system has generated promising efficacies, although its long‐term benefits remain unclear. In addition, owing to the development of immunoresponse to certain cancers, the response after tumor implantation tends to follow the biological features of the tumors. Therefore, a further understanding of the postoperative immunity changes is needed to enable development of related treatments that improve survival rates of patients with solid cancers. Overall, we have highlighted surgical trauma‐induced immunity dysfunction, which is a critical point for reducing postoperative cancer metastasis, especially for cancer types that require extensive surgical resection during removal of local tumors.

## CONFLICT OF INTEREST

The authors declared no conflict of interest.
